# Physical fitness and motor ability parameters as predictors for skateboarding performance: A logistic regression modelling analysis

**DOI:** 10.1371/journal.pone.0296467

**Published:** 2024-02-08

**Authors:** Aina Munirah Ab Rasid, Rabiu Muazu Musa, Anwar P. P. Abdul Majeed, Ahmad Bisyri Husin Musawi Maliki, Mohamad Razali Abdullah, Mohd Azraai Mohd Razmaan, Noor Azuan Abu Osman

**Affiliations:** 1 Centre for Fundamental and Continuing Education, Department of Credited Co-Curriculum, Universiti Malaysia Terengganu, Kuala Nerus, Malaysia; 2 School of Robotics, XJTLU Entrepreneur College (Taicang), Xi’an Jiaotong-Liverpool University, Suzhou, China; 3 Defense Fitness Academy, Universiti Pertahanan Nasional Malaysia (UPNM) Sungai Besi Camp, Sungai Besi, Kuala Lumpur; 4 Faculty of Health Science, Universiti Sultan Zainal Abidin, Kuala Nerus, Terengganu, Malaysia; 5 Innovative Manufacturing, Mechatronics and Sports Laboratory, Faculty of Manufacturing and Mechatronic Engineering Technology, Universiti Malaysia Pahang, Pekan, Pahang, Malaysia; 6 Centre for Applied Biomechanics, Department of Biomedical Engineering, Faculty of Engineering, University of Malaya, Kuala Lumpur, Malaysia; Università degli Studi di Milano: Universita degli Studi di Milano, ITALY

## Abstract

The identification and prediction of athletic talent are pivotal in the development of successful sporting careers. Traditional subjective assessment methods have proven unreliable due to their inherent subjectivity, prompting the rise of data-driven techniques favoured for their objectivity. This evolution in statistical analysis facilitates the extraction of pertinent athlete information, enabling the recognition of their potential for excellence in their respective sporting careers. In the current study, we applied a logistic regression-based machine learning pipeline (LR) to identify potential skateboarding athletes from a combination of fitness and motor skills performance variables. Forty-five skateboarders recruited from a variety of skateboarding parks were evaluated on various skateboarding tricks while their fitness and motor skills abilities that consist of stork stance test, dynamic balance, sit ups, plank test, standing broad jump, as well as vertical jump, were evaluated. The performances of the skateboarders were clustered and the LR model was developed to classify the classes of the skateboarders. The cluster analysis identified two groups of skateboarders: high and low potential skateboarders. The LR model achieved 90% of mean accuracy specifying excellent prediction of the skateboarder classes. Further sensitivity analysis revealed that static and dynamic balance, lower body strength, and endurance were the most important factors that contributed to the model’s performance. These factors are therefore essential for successful performance in skateboarding. The application of machine learning in talent prediction can greatly assist coaches and other relevant stakeholders in making informed decisions regarding athlete performance.

## Introduction

Skateboarding has gained popularity as an extreme sport among young people, despite the associated risk of injury. The sport’s appeal can be attributed to its ability to encourage self-expression, foster creativity, and embrace a freestyle approach without rigid rules [1]. Furthermore, the inclusion of skateboarding as one of the sports in the 2020 Summer Olympics in Tokyo aimed not only to attract young spectators but also to provide skateboarders with a productive platform to showcase their talents [2]. The increasing pressure to excel in international sports, particularly following the global lockdown two years ago, highlights the importance of identifying and nurturing talents to effectively represent the nation in sporting events. This underscores the significance of developing and supporting aspiring skateboarders to help them reach their full potential and contribute to their country’s success in the field of sports [3].

The rapid advancement of technology in the domain of data collection, storage, and analysis has profoundly catalyzed the implementation of data-driven decision-making across different domains notably within the realm of sports [4, 5]. Through the utilization of wearable sensors, camera capture techniques, and questionnaires, comprehensive information systems can be developed, allowing coaches, stakeholders, and practitioners the capacity to acquire detailed insights into athletes’ physical, technical, and psychological attributes [6]. These datasets prove invaluable in the identification and selection of high-performing athletes based on their unique attributes and abilities [7]. However, despite the availability of such system, coaches often still rely on observation-based decision-making, utilizing their knowledge and experiences to identify athletes who they believe possess the criteria to excel in sports [8]. However, such naturalistic decision-making processes are susceptible to errors, including biased selections influenced by personal preferences, and may not consistently yield optimal outcomes [9–11].

Numerous studies have underscored the significance of motor skills and fitness-related variables in enhancing athletic performance and mitigating the risk of injuries [12]. For instance, a recent investigation by Garciá-Pinillos et al. (2017) [13] elucidated that athletes with higher levels of motor skills and fitness ability tend to demonstrate superior on-field performance and heightened endurance, thereby presenting a lower onset of fatigue Skateboarding athletes rely heavily on the use of their lower body limbs to execute tricks with ease and precision. Consequently, developing lower and core body strength, static and dynamic balance controls could be crucial components for improving skateboarding performance. Additionally, research has shown that an athlete’s fitness and motor skills play a vital role in the learning process of new sports skills, helping to efficient acquisition of new techniques [14–16].

Generally, it could be speculated that combining motor skills and fitness-related variables can be a valuable tool for identifying talent, improving athletic performance, and reducing the risk of injury in a variety of sports, including skateboarding.

Additionally, machine learning, a subfield of Artificial Intelligence (AI), is dedicated to the development of applications or systems capable of autonomously learning from existing data and enhancing their accuracy through experiences without explicit programming [15, 17]. The algorithms employed in machine learning analysis are designed to identify patterns and characteristics in large datasets to facilitate decision making and prediction, leading to the creation of new data [18]. In essence, machine learning revolves around empowering machines to proficiently manage extensive datasets [19]. This technique not only expedites the decision-making process for coaches but also significantly reduces the time required.

Logistic Regression-based machine learning stands out as one of the most effective technique for addressing classification problems, renowned for its robustness in elucidating the interplay between the dependent and independent variables through interpretable coefficients. Moreover, it tends to exhibit greater resilience against overfitting issues [20, 21]. The application of this algorithm has consistently proven to be a viable approach in identifying potential athletes based on specific parameters, thereby ensuring a bias-free athlete selection process [22, 23]. While all athletes can improve their skills and fitness through training, selecting the most talented individuals for competition representation reduces time and costs. This, in turn, enables both coaches and athletes to concentrate on a tailored training regimen that best aligns with their unique requisites.

Therefore, this paper aims to explore the potential of machine learning in predicting skateboarding athletes’ class based on their fitness and motor skills consisting of the stork balance test, star excursion balance test (SEBT), countermovement jump (vertical jump) tests, single-leg wall sits tests, standing broad jump tests as well as plank and sit-up tests. Specifically, we intend to achieve the following objectives:

- Develop a logistic-based machine learning pipeline for predicting potential skateboarding athletes.
- Test the model against unseen or fresh dataset to examine its effectiveness towards predicting future potential.
- Identify the variables that highly contribute to model performance via feature importance to determine the contribution of each fitness and motor skill variable to overall accuracy of the model.

## Materials and methods

### Participants

A total of 45 male skateboarders were randomly selected from different skate parks along the East Coast of Malaysia. They were given a brief introduction to the experimental procedures and willingly committed to providing their full cooperation throughout the entire process. It is worth noting that these skateboarders engaged in the activity solely for recreational purposes, none of the participants had affiliations with either governmental or private skateboarding clubs. Specialized training was not administered to the participants before the data collection phase, which commenced on February 4^th^ and concluded on March 5^th^, 2022, evening (5–7 pm).

### Motor skill and fitness measurements

The participants underwent specific bio-fitness assessments to test their physical strengths in relation to the demands of skateboarding. The bio-fitness assessment protocol encompassed seven distinct tests, which include the stork balance, star excursion balance test (SEBT), vertical jump, single-leg wall sits, standing broad jump, plank, and sit-up tests. Before commencing the assessments, participants received comprehensive explanations of the experiment and underwent health screenings to ensure they were in good physical condition and free from injuries, both current and historical. To reduce the risk of injuries, participants were advised to engage in three minutes of warm-up and stretching exercises in preparation for the tests.

The assessment tests were divided into separate sections and administered sequentially. Between each test, participants were granted a five-minute break to rest and rehydrate. The stork balance test (flamingo test) evaluated participants’ static balance abilities [24]. Initially, the athletes were instructed to assume a comfortable stance with their feet positioned shoulder-width apart. Subsequently, they were directed to raise their right legs, positioning the soles of their right feet against the knees of their left legs. Upon the commencement of the stopwatch, athletes were immediately instructed to elevate their left feet onto their tiptoes and maintain this posture until they exhibited signs of instability, at which point the stopwatch was promptly halted. The test was repeated for the left leg.

Meanwhile, the SEBT assessed their dynamic balance capabilities [25]. The test began with the preparation of a marked, non-slip surface, featuring a star-shaped configuration created by four intersecting 120 cm-long tapes. Subsequently, athletes were asked to stand barefoot at the centre of the star shape with hands on their hips. The athletes were directed to extend their right foot in each direction, lightly touching the marked lines before returning to their starting position. This process was repeated three times for each leg, and the researcher recorded the distances rounded to the nearest 0.5 cm. It’s crucial to note that the test would be considered invalid if subjects couldn’t return to their starting positions or displayed instability.

Further, to assess leg power, athletes or skateboarders were asked to perform a Sargent jump test. The test required the use of a Vertec apparatus, which was positioned on a flat surface, allowing sufficient space for safe jumping and landing. Skateboarders were initially given a demonstration and then instructed to stand with their feet close together, extending one arm upward until they touched the vane. The vane’s height was adjustable based on the skateboarder’s height and arm extension. After setting the vane at the appropriate height, skateboarders were directed to jump as high as possible, attempting to touch the highest point of the vane. The score was determined by the difference between their standing reach height and their jump height [26].

The next leg test assessed the leg strength of the skateboarding athletes, a crucial factor in enhancing their performance [27]. Specifically, a single-leg wall sit test was conducted. The skateboarders were instructed to stand with their feet shoulder-width apart and their backs against the wall. Subsequently, they were guided to slowly lower themselves down the wall, mimicking the posture of sitting in a chair with their knees bent at a 90-degree angle. If necessary, skateboarders were allowed to adjust their foot placement. Regarding hand positioning, skateboarders could choose to place their hands on their hips or cross them over their chests. The researcher initiated a stopwatch when skateboarders raised one of their legs off the ground, stopping it when they were no longer able to maintain the position, causing their legs to touch the ground. After a brief pause, the same procedure was repeated for the other leg. Upon completing the assessments for all skateboarders, the researcher recorded the elapsed time for each leg.

For testing the explosive leg power of the skateboarders, h the standing broad jump (SBJ) test was used which incorporated a long jump landing mat [28]. The athletes were asked to position their legs at the starting point on the mat with their feet slightly apart. Following this, the athletes were directed to bend their knees and swing their arms to generate momentum before initiating the jump. When prepared, the athlete endeavoured to jump as far as possible, ensuring a stable landing on both feet without falling backwards. The researcher marked the distance achieved by the athlete and recorded it in meters. This process was repeated for a total of three trials, with the athlete executing the jumps accordingly.

For the evaluation of the skateboarders’ core muscle strength, a plank fitness test was employed. The plank fitness test is a straightforward assessment, which can also serve as a fitness exercise for enhancing core strength [29]. The skateboarders were asked to assume a prone position, facing the floor. They were then guided to support their upper bodies with their elbows, forearms, and straightened legs, with their body weight balanced on their toes. Once in the correct position, the researcher initiated the timing process. Skateboarders were required to maintain a straight back with their heads facing the ground, and the test concluded when participants could no longer sustain this position, resulting in lowered hips. Upon completing the assessment for all skateboarders, the researcher recorded the time for each participant at the plank position.

Finally, the sit-up test was employed to measure abdominal muscle strength and endurance [30]. To begin, a flat and clean surface was used with a stopwatch to monitor the athlete’s performance. Subsequently, the athletes were instructed a position lying down with their knees flexed and their feet positioned about 30 cm from their buttocks. To ensure stability, a partner was directed to place their knees on the athlete’s feet, preventing them from lifting off the ground. Meanwhile, the athlete’s hands were crossed over their chest. Once the athlete was prepared, the researcher initiated the stopwatch and counted the number of sit-ups the athlete could complete within one minute. Upon completion, the researcher recorded the total number of sit-ups performed.

It is important to note that all data underwent standardization to ensure that each variable contributed equally to the analysis, thus eliminating potential biases arising from differences in the measurement scales of the evaluated variables. Furthermore, the data met the criteria for a normal distribution based on the Shapiro-Wilks test, with a p-value exceeding 0.05.

### Skateboard skill performance evaluation

Following the completion of the bio-fitness assessments, athletes were afforded additional rest time before their skateboarding skills were assessed. The study centred on the evaluation of five fundamental skateboarding tricks: Ollie, Nollie, Frontside 180, Pop-Shuvit, and Kickflip, all of which had been extensively scrutinized and studied in previous literature [3, 31–35].

Each participant was granted three opportunities to execute each trick under the watchful eyes of the researchers. A successful performance was recorded when an athlete executed the trick or landed securely on the skateboard without any stumbles. Conversely, if an athlete failed to execute the trick or land on the skateboard successfully, the performance was deemed unsuccessful. These observations were made based on the judging criteria and scoring systems from the 2020 Olympic Games established by World Skate in collaboration with the International Olympic Committee (IOC). The judging criteria consisted of five general elements: difficulty and variety of performed tricks, quality of execution, use of the course and individual obstacles, flow and consistency, as well as repetition. For this study, since the participants were tasked with performing only the five specified tricks, the focus was on the quality of execution as per the World Skate general judging criteria. This encompassed the assessment of how well the tricks were performed, whether they were executed successfully or not, and the athlete’s ability to consistently land tricks of a certain difficulty level. Execution also considered various aspects such as style, speed, distance, and height throughout the entire execution of the trick, from start to finish. It was essential that the landing was performed smoothly, continuously without interruptions, and with full control throughout the entirety of the athlete’s performance [36, 37]. The researchers evaluated the skateboarders’ trick performances in real-time through observation, aided by the input of other skateboarders present who provided their judgments. The statistical analysis used the percentage of successful tricks out of the total attempted to determine performance scores for each trick (tricks’ performance %) [38].

### Clustering

K-means clustering is an unsupervised learning algorithm employed to partition datasets into k-distinct clusters. It begins by selecting *k* centroids or centres for these clusters and proceeds by iteratively assigning each data point to the closest centroid [39–41]. The centroid is then updated to represent the average of the points assigned to it, and this process continues until the centroid no longer undergoes significant movement. The primary objective is to minimise the total distance between the data points and assigned centroids. In the context of this study, k-means clustering was utilized to segment a set of data into subgroups, creating *k* non-overlapping clusters, with each data point associated with a single cluster. The primary aim is to enhance the similarity of the data points within each cluster while preserving the distinctions between data points belonging to different clusters. This clustering method relies on the Euclidean distance metric to determine the formation of clusters.

### Logistic regression model development

A logistic regression model (LR) was employed to evaluate the effectiveness of the models in classifying two groups of skateboarders derived from the cluster analysis. It is important to note that before applying LR, the data in the study underwent a process called standardization. Standardization was carried out to ensure that all selected variables were on a common scale, ranging from 0 to 1. This process helps prevent certain variables with larger numerical ranges from disproportionately influencing the model. Standardization involves transforming the values of each variable so that they have a mean (average) of 0 and a standard deviation of 1. This process makes it easier for the model to work with the data, as it removes any potential bias introduced by the different scales of the variables. Standardizing the data ensures that each variable contributes equally to the modelling process. To validate the performance of the model, a five-fold stratified cross-validation technique was used [42]. This technique is particularly useful when dealing with imbalanced datasets or minority classes, as it ensures that each fold of data has a representative mix of various classes present. Subsequently, the average performance across all folds is computed. This approach is preferred to address overfitting, a common challenge when training models on a specific subset of data.

The data was divided into a 70:30 ratio, with 70% for training and 30% for testing [43, 44]. A total number of 29 skaters were utilized to train the model, whilst the remaining 13 observations were used independently to assess the classifiers’ predictability in identifying HPS and LPS. For the development of the LR model, the Pycaret libraries were invoked using the Spyder IDE. Additionally, other statistical analyses were carried out using the add-in software and Orange Canvas version 3.4.0

### Model evaluation

In this study, we conducted a thorough assessment of the LR model’s performance, employing a diverse set of performance metrics. These metrics encompass key indicators, including classification accuracy (ACC), the area under the curve (AUC), Recall, precision (PREC), F1 score, Kappa, and Matthew’s correlation coefficient (MCC). To elaborate further, ACC is the ratio of correctly classified instances to the total number of instances. AUC on the other hand, assesses the model’s ability to differentiate between classes and quantifies its performance in separating the two classes [45]. Besides, recall measures the proportion of actual positive instances correctly predicted as positive, while PREC computes the ratio of the number of correct positive predictions to the total number of positive predictions [46]. Moreover, the F1 score is the harmonic mean of precision and recall and measures the average proportion of accuracy for both positive and negative instances [47]. Kappa assesses how closely the classifier’s instances match the ground truth data, accounting for the expected accuracy of a random classifier. Similarly, MCC is a discrete version of Pearson’s correlation coefficient, with a range of 1 to 1, where 1 indicates a completely correct binary classifier and −1 indicates the opposite [48]. It measures the quality of binary classification and gauges the performance of the classification models. Finally, the confusion matrix allows for the observation of correctly classified and misclassified instances that occur between the defined classes [49–51]. Furthermore, we conducted a sensitivity analysis using feature importance plots to delve deeper into the motor skill and fitness performance variables contributing to the model’s accuracy in the study [52]. Collectively, these performance metrics provide a comprehensive evaluation of the LR model’s effectiveness in addressing the classification problems in the present investigation.

## Results

Table 1 presents the descriptive statistics for the measured variables. This table provides a comprehensive overview of several key aspects, including the participant count, the variables of the study, and the corresponding mean values and standard deviations.

**Table 1:** Descriptive statistics of the study variables.

Variable	N	Mean	Std. dev
Age	45	23.089	5.405
Skate Experience (yr)	45	6.759	6.331
Height (m)	45	1.672	0.0072
Weight (kg)	45	60.802	12.413
Stork Balance (s)	45	23.082	10.870
Single-leg wall sit (s)	45	32.184	16.952
Vertical Jump (m)	45	0.955	0.245
SEBT normComposite (%)	45	73.636	7.272
Plank (s)	45	65.149	27.453
Standing Broad Jump (m)	45	2.246	0.306
Sit-up (max no sit up/min)	45	27.467	5.476
Tricks’ Performance (%)	45	0.686	0.238

**Fig 1:**
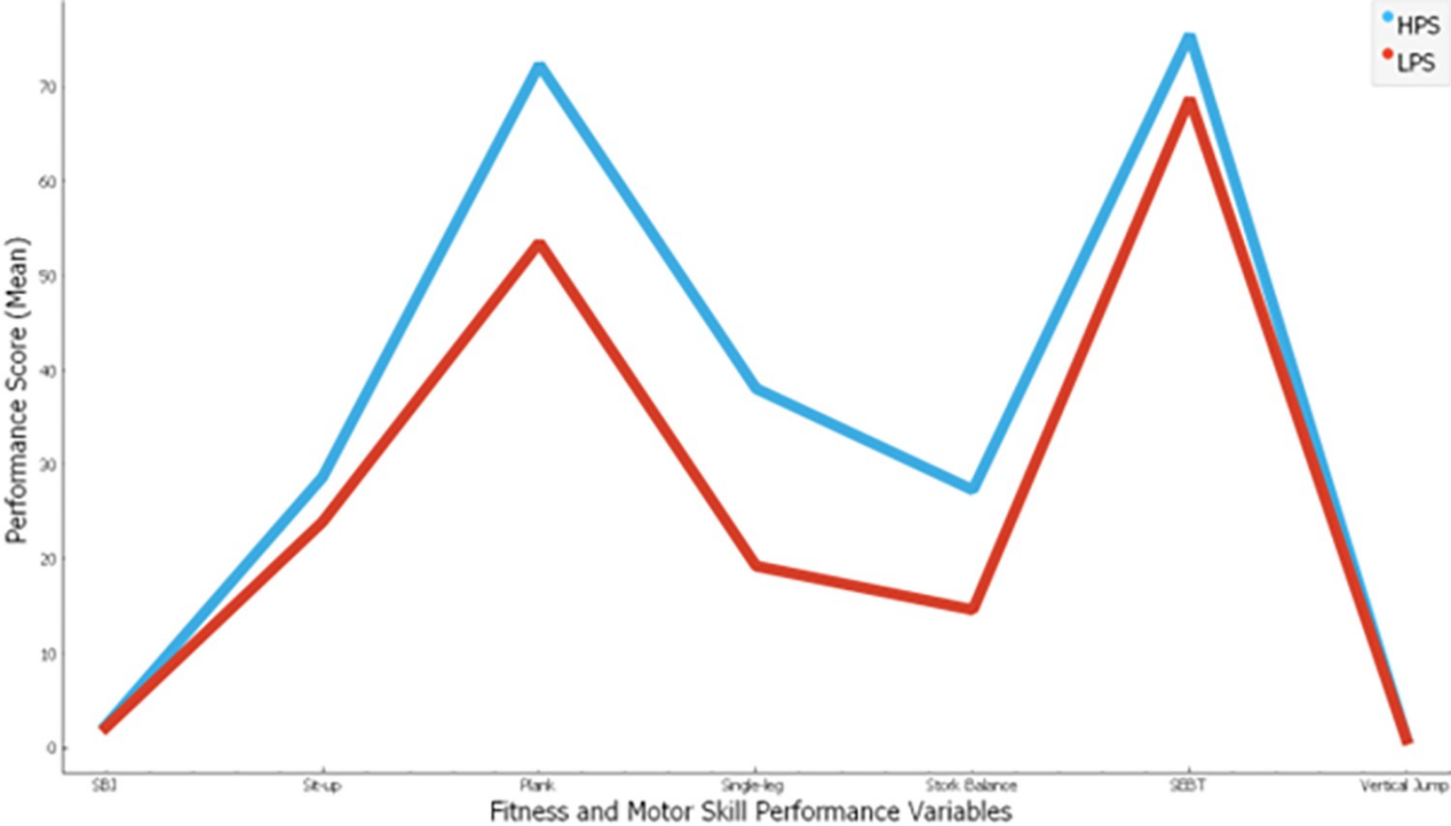


Fig 1 illustrates the outcomes of the k-means clustering analysis, which categorizes each skateboarder according to their performance levels. This clustering process is rooted in the assessment of performance across all bio-fitness components we examined. The illustration of the clustering results distinctly reveals the emergence of two well-defined groups: high-potential skaters (HPS) and low-potential skaters (LPS), characterized by their skateboarding performance scores. Our analysis classifies 31 out of the total 45 participants as HPS, while the remaining 14 participants fall into the LPS category. Notably, it is evident that the HPS group consistently outperformed the LPS group across all the variables scrutinized in our study.

Table 2 provides a summary of the logistic regression model’s performance in predicting the two distinct groups of the skateboarders.

**Table 2:** Performance evaluation of the logistic regression model for predicting high and low potential skateboarders from fitness and motor skill variables.

	Accuracy	AUC	Recall	Prec.	F1	Kappa	MCC
Mean	0.900	1.000	0.900	0.866	0.833	0.781	0.815
Std	0.133	0.000	0.200	0.266	0.211	0.278	0.232

The mean score in the table reveals that the model achieved an accuracy of 90%, indicating an excellent prediction of the skateboarder classes. Moreover, the Area Under the Curve (AUC) value stands at 1.000, indicating a flawless ability to predict skateboarder classes, reflecting a perfect model fit.

The F1 score, a weighted amalgamation of Precision and Recall, reveals noteworthy values of 0.866 and 0.900, respectively. These scores denote the model’s proficiency in correctly predicting over 85% of positive cases and accurately identifying 90% of the actual positive classes. The Area Under the Curve (AUC) was 1.000, which signifies perfect modelling in predicting skateboarder classes. The F1 or F Score is a weighted average score that correlates between Precision and Recall. The table reports that the Precision and Recall scores were 0.866 and 0.900, respectively. These scores demonstrate that the model predicted more than 85% of positive cases and correctly identified 90% of the actual positive classes. The Kappa score obtained by the model was 0.781, which indicates good reliability, while the Matthew’s Correlation Coefficient (MCC) demonstrated an excellent prediction of 0.815. Overall, these findings suggest that the logistic regression model performed well in predicting the two groups of skateboarders.

**Fig 2:**
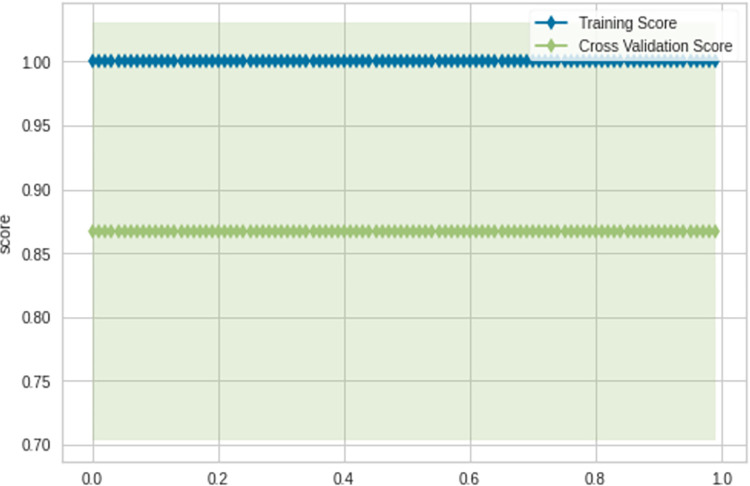


Fig 2 illustrates the training and cross-validation scores of the model as applied to the test dataset. Notably, the training score registers at 1.00, while the cross-validation score is slightly lower at 0.85. This divergence between training and cross-validation scores is a natural consequence of the data partitioning for training and testing purposes.

This phenomenon can be attributed to the initial inclusion of potential noises and errors within the training data, as suggested by previous research [53]. The process of cross-validation effectively fine-tuned the model, yielding a prediction accuracy score of 85%. This outcome underscores the efficiency and robustness of the model, indicating that it has been finely calibrated to provide the best fit for the data.

**Fig 3:**
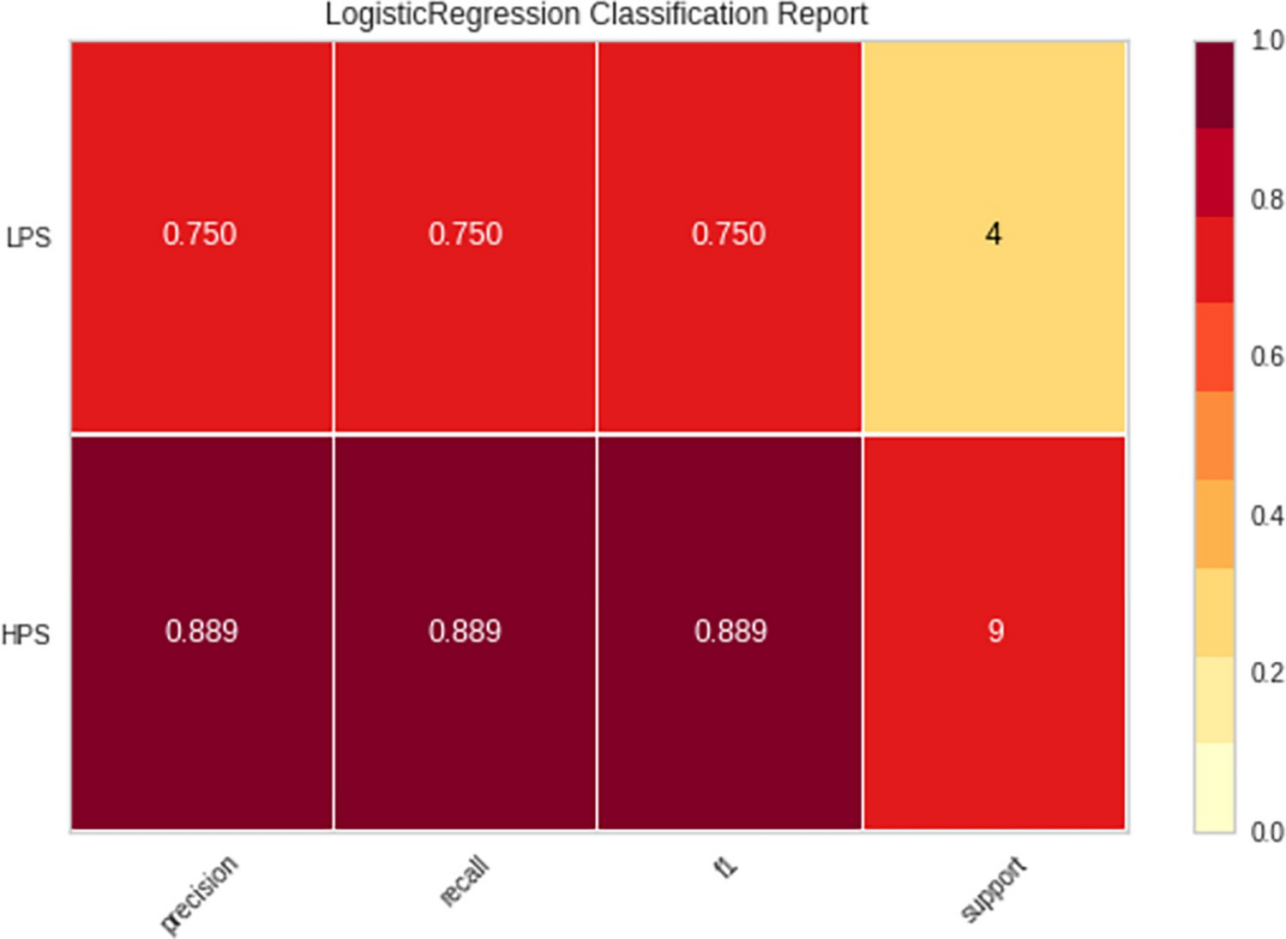


Fig 3 highlights the results of the logistic regression analysis for the classification of skateboarders after cross-validation. It is important to note that cross-validation is a process of validating the effectiveness of the formulated model on untrained data. In this stage, the new sample of untrained data was tested using the previously formulated logistic regression model to examine the accuracy of the model developed. It is apparent that the F1, precision and Recall scores reduce as cross-validation takes place. For HPS, the F1 score is similar to Precision and Recall at 0.889 while LPS has 0.750 for all the aforementioned variables. These observations reflect the model’s performance when subjected to new, untrained data and underscore the distinctions in its predictive power for the two distinct skateboarder categories.

**Fig 4:**
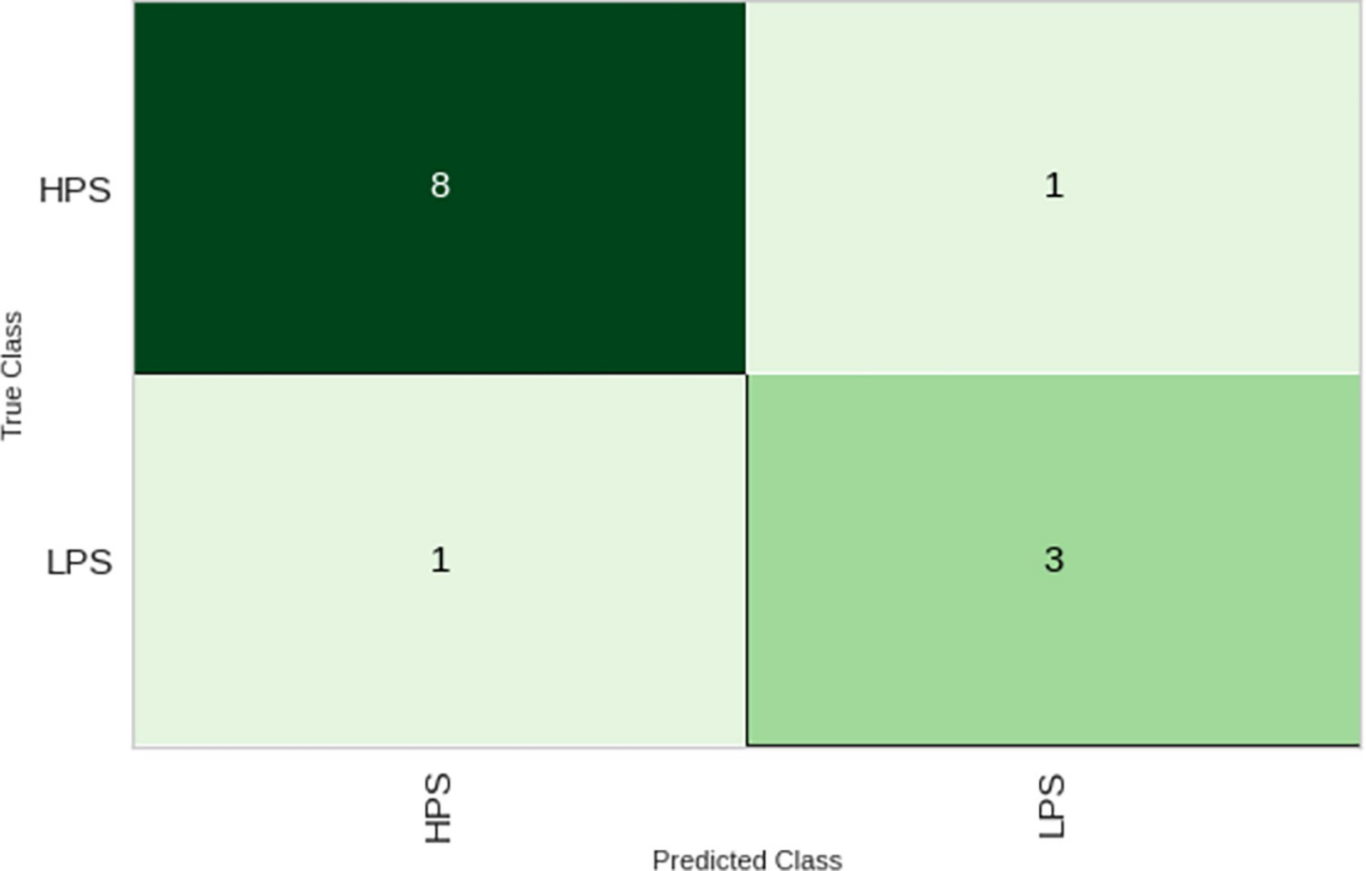


Fig 4 visually presents the confusion matrix of the developed model following the completion of cross-validation. This analytical technique was implemented to assess the model’s proficiency in accurately classifying the skateboarder categories.

The results from the confusion matrix reveal that the model adeptly classified the high-potential skaters (HPS), with 8 out of 9 individuals correctly categorized and only 1 misclassification. Similarly, the model displayed sound performance in the identification of low-potential skaters (LPS), with 3 out of 4 being correctly classified and only one instance of misclassification within the LPS group.

Overall, it is evident that the model demonstrated worthy performance in the classification task against the test data, despite the somewhat limited number of observations available for evaluation.

### Sensitivity analysis: Identification of most essential variables towards the model accuracy

**Fig 5:**
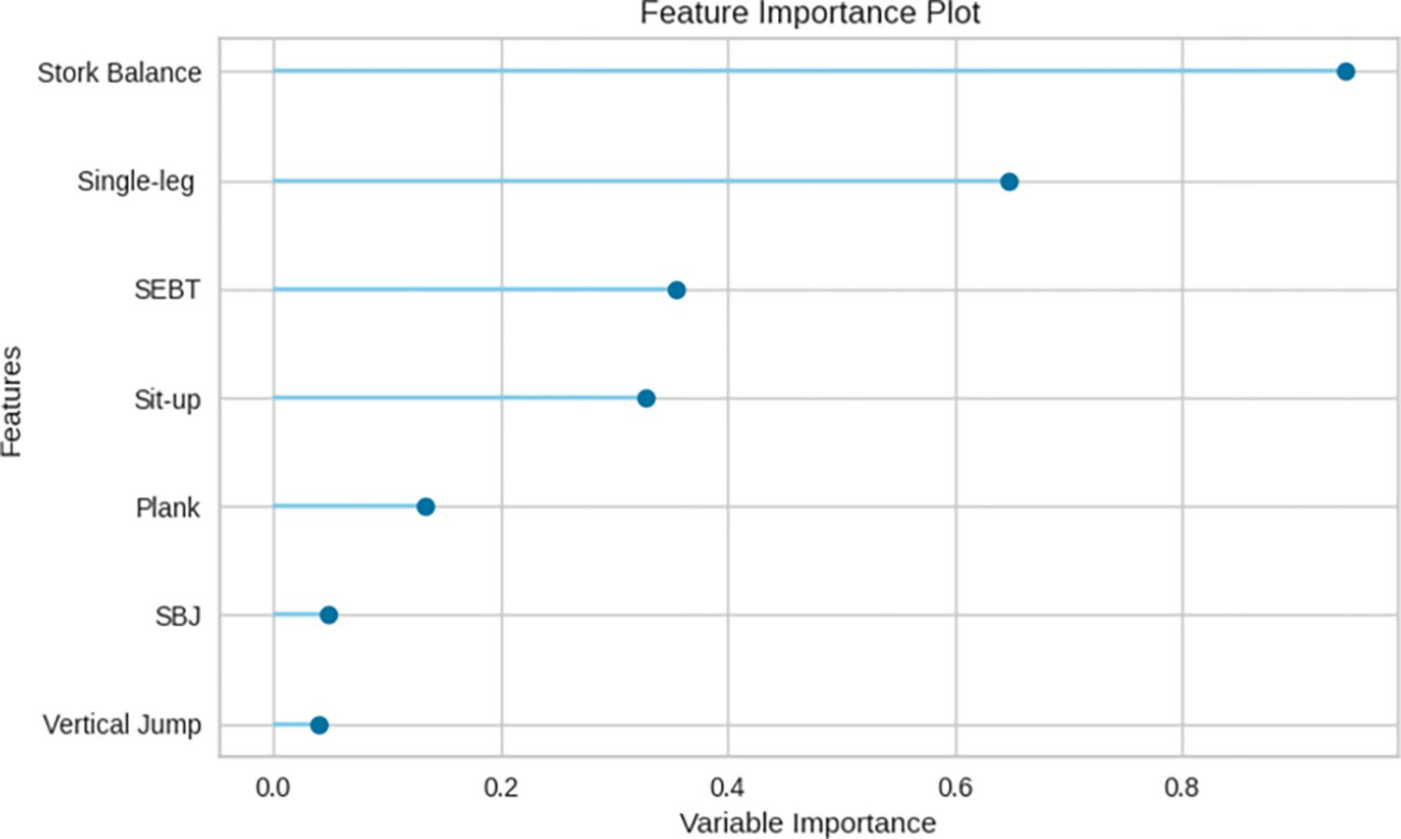


Fig 5 offers a visual representation of how various variables contribute to the performance of the model pipeline, as indicated by the feature importance plots. This graphical analysis reveals distinctive patterns in the significance of different factors.

From the figure, it is apparent that four out of the seven assessed fitness and motor skill variables namely, stork balance, single leg wall sits, SEBT, and sit-up play a more substantial role in determining the model’s overall performance. This underscores their pivotal importance in the context of skateboarding, suggesting that they significantly influence the sport’s successful execution.

Conversely, the plank test, SBJ, and vertical jump exhibit relatively lower levels of importance in the context of the model’s overall performance. These findings shed light on the varying degrees of influence that different variables exert on the model’s predictive power, offering valuable insights into the factors contributing to skateboarding performance.

## Discussion

The findings of this study reveal that the selected fitness and motor skill variables under consideration play a significant role in predicting and classifying the performance of skateboarders, distinguishing between HPS and LPS based on their skateboarding trick performance scores. The application of k-means clustering analysis, as depicted in Fig 1, allowed us to cluster athletes based on their measured fitness and motor abilities, which include static balance, dynamic balance, leg peak power, leg strength, explosive leg power, core muscle strength, as well as abdominal muscle strength and endurance.

Moreover, the profile plot presented in the figure underscores that the mean performance of HPS surpasses that of LPS across all the mentioned performance variables.

We have thoroughly investigated the LR model pipeline’s performance in classifying skateboarder categories, demonstrating that it excels in distinguishing HPS from LPS. The model achieved a noteworthy mean accuracy of 90% in the training dataset, signifying its excellence in predicting skateboarder categories. The model’s AUC reached a perfect score of 1.000. attesting to the accuracy of its predictions. Notably, both Precision and Recall scores were impressive, with values of 0.8667 and 0.9, respectively, highlighting its ability to make positive predictions and correctly identify actual positive classes. These results are summarized in Table 2.

Furthermore, our findings indicate that the LR model performs effectively on unseen datasets, with only one misclassification observed in both HPS and LPS groups. This suggests that the model can predict the performance of new skateboarders even with a relatively limited number of observations, aligning with prior research that underscores the LR model’s efficacy in solving classification challenges in the realm of sports [21, 54].

In our sensitivity analysis, we have identified certain physical fitness and motor skill variables that exhibit greater importance in predicting skateboarding performance. Particularly, the core muscle strength, explosive leg power, and leg peak power were found to have less influence, as illustrated in Fig 5. On the contrary, attributes like static balance, dynamic balance, leg strength, and abdominal muscle strength and endurance played an influential role in classifying performance levels. These variables have been well-documented in the literature as critical factors in athletic performance, talent identification, and injury prevention [55].

Balancing capabilities, both static and dynamic, are paramount for executing precise tricks and achieving ideal landing postures in skateboarding. This discovery aligns with prior research that underscores the necessity for skateboarders to maintain balance during rapid transitions to achieve the desired final stance [56].

Physical fitness is a crucial facet of an athlete’s performance. Tests such as single-leg wall sit and sit-ups evaluate the lower body and abdominal muscle strength and endurance, which are integral not only for enhancing sports skill efficiency but also for building endurance and delaying fatigue [57]. For skateboarders, lower body strength is essential for generating the force needed to perform complex tricks effectively. Abdominal muscle strength and endurance, on the other hand, are vital for maintaining balance and stability during trick execution. It allows skateboarders to execute manoeuvres smoothly, even under twisting and turning conditions [38, 58].

## Conclusion and future direction

The results of this investigation emphasize the pivotal role played by specific fitness and motor ability variables, including static and dynamic balance, abdominal muscle strength and endurance, and lower limb strength, in determining the performance class of skateboarders. In addition, the application of machine learning algorithms, specifically logistic regression, has proven its effectiveness in accurately predicting the classification of skateboarders based on the chosen performance variables. The application of machine learning techniques holds considerable significance, as it empowers coaches to identify potential athletes in skateboarding through a concise set of essential physiological measurements, offering a streamlined and cost-effective approach to talent identification programs.

The study further highlights the potency of machine learning algorithms in quantifying and distinguishing between high-potential and low-potential athletes by utilizing a predefined set of pertinent performance variables within the realm of skateboarding. However, it is imperative to acknowledge the study’s limitations, outstandingly its constrained sample size and specific demographic characteristics. These constraints hinder the generalization of findings to broader categories of skateboarder participation. Consequently, the authors advocate for the extension of these proposed methods to cater to various sports and disciplines, enabling the identification of talent and the classification of performance capabilities among athletes in a more comprehensive manner.

## Supporting information

S1 Datasets (XLSX)
